# Mean Arterial Pressure (MAP) Trial: study protocol for a multicentre, randomized, controlled trial to compare three different strategies of mean arterial pressure management during cardiopulmonary bypass

**DOI:** 10.1186/s13063-024-07992-3

**Published:** 2024-03-15

**Authors:** Alessandra Francica, Gina Mazzeo, Antonella Galeone, Daniele Linardi, Livio San Biagio, Giovanni Battista Luciani, Francesco Onorati

**Affiliations:** Division of Cardiac Surgery, Department of Surgery, Dentistry, Paediatrics and Gynaecology, University of Verona, Piazzale Stefani 1, 37126 Verona, Italy

**Keywords:** Cardiopulmonary bypass, Mean arterial pressure, Cardiac surgery, Randomized controlled trial

## Abstract

**Background:**

One of the main goals of cardiopulmonary bypass (CPB) is targeting an adequate mean arterial pressure (MAP) during heart surgery, in order to maintain appropriate perfusion pressures in all end-organs. As inheritance of early studies, a value of 50–60 mmHg has been historically accepted as the “gold standard” MAP. However, in the last decades, the CPB management has remarkably changed, thanks to the evolution of technology and the availability of new biomaterials. Therefore, as highlighted by the latest European Guidelines, the current management of CPB can no longer refer to those pioneering studies. To date, only few single-centre studies have compared different strategies of MAP management during CPB, but with contradictory findings and without achieving a real consensus. Therefore, what should be the ideal strategy of MAP management during CPB is still on debate. This trial is the first multicentre, randomized, controlled study which compares three different strategies of MAP management during the CPB.

**Methods:**

We described herein the methodology of a multicentre, randomized, controlled trial comparing three different approaches to MAP management during CPB in patients undergoing elective cardiac surgery: the historically accepted “standard MAP” (50–60 mmHg), the “high MAP” (70–80 mmHg) and the “patient-tailored MAP” (comparable to the patient’s preoperative MAP). It is the aim of the study to find the most suitable management in order to obtain the most adequate perfusion of end-organs during cardiac surgery. For this purpose, the primary endpoint will be the peak of serum lactate (Lmax) released during CPB, as index of tissue hypoxia. The secondary outcomes will include all the intraoperative parameters of tissue oxygenation and major postoperative complications related to organ malperfusion.

**Discussion:**

This trial will assess the best strategy to target the MAP during CPB, thus further improving the outcomes of cardiac surgery.

**Trial registration:**

NCT05740397 (retrospectively registered; 22/02/2023)

## Administrative information

Note: the numbers in curly brackets in this protocol refer to SPIRIT checklist item numbers. The order of the items has been modified to group similar items (see http://www.equator-network.org/reporting-guidelines/spirit-2013-statement-defining-standard-protocol-items-for-clinical-trials/).

**Table:** 

Title {1}	Mean Arterial Pressure (MAP) Trial: study protocol for a multicenter, randomized, controlled trial to compare three different strategies of mean arterial pressure management during cardiopulmonary by-pass
Trial registration {2a and 2b}.	NCT05740397 (retrospectively registered on ClinicalTrials.gov; 22/02/2023): https://clinicaltrials.gov/ct2/show/NCT05740397?term=NCT05740397&draw=2&rank=1
Protocol version {3}	Version 2 of 27-03-2021
Funding {4}	The project does not receive any source and any other type of financial, material or other supports
Author details {5a}	AF. GM, AG. DL. LSB. GBL. FO. Division of Cardiac Surgery; Department of Surgery, Dentistry, Paediatrics and Gynaecology; University of Verona; Italy.
Name and contact information for the trial sponsor {5b}	No external sponsor.
Role of sponsor {5c}	No external sponsor.

## Introduction

### Background and rationale {6a}

Most of cardiac surgery operations require a bloodless operating field and a steady heart (i.e. cardioplegic arrest). To ensure the perfusion of peripheral organs during the cardioplegic arrest time, the cardiopulmonary bypass (CPB) is installed. The CPB is a biomedical device, also named Heart-Lung machine, that, through a circuit, filtrates and oxygenates the venous blood of the patient, giving it back as arterial blood through a mechanical pump. The target blood flow during CPB is traditionally determined according to body surface area (BSA) and temperature. Under moderate hypothermia-to-normothermic conditions, the pump flow rate is set between 2.2 and 2.8 l/min/m^2^ by the majority of perfusionists [1]. Therefore, the CPB provides continuous blood flow, so the pressure produced is a “mean arterial pressure” (MAP). The MAP can be modulated by vasoactive and/or anaesthetic drugs, in order to maintain an appropriate perfusion pressures in all end-organs, particularly the kidneys, the brain and the gastrointestinal tract, avoiding tissue hypoperfusion and hypoxia. The latter, indeed, may occur in the postoperative period as organ damage: cerebral ischaemia and consequently transitory or permanent neurological injury [2], renal ischaemia and consequently acute kidney injury [3, 4], or gastrointestinal ischaemia [5, 6]. Therefore, the hypoperfusion during CPB could directly affect the morbidity and mortality of cardiac surgery. Consequently, the management of MAP acquires a crucial role. A value of 50–60 mmHg has been historically accepted as the “gold standard” MAP, according to the pioneering animal experiments and investigations of the dawn of cardiac surgery (around ‘50s) [2, 7–11]. However, in the last decades, the CPB has remarkably changed thanks to the evolution of technology and the availability of new biomaterials. Therefore, as highlighted by the latest European Guidelines [1], the current management of CPB can no longer refer to those pioneering studies. To date, only few authors focused their attention on the correlation between MAP values during CPB and cardiac surgery outcomes. Vedel et al. [12] compared the “high-target” MAP (70–80 mmHg) to “standard” MAP (50–60 mmHg) in terms of new postoperative cerebral injuries. They reported a higher incidence of stroke (7.0% vs 1.1%; *P*=0.09) and mortality (4.1% vs 0%; *P*=0.06) in the “high-target” MAP population compared to the “low-target” group. Conversely, Gold et al. [13] reported a reduction (from 12.9 to 4.8%) of major cardiac and neurologic events in patients treated with higher MAP compared to patients treated with the “standard” MAP during coronary artery bypass graft surgery (CABG). However, the abovementioned studies are both from a single institution, with small and selected sample sizes (i.e. CABG). Finally, Charleson et al. [14] compared the “high MAP” (80 mmHg) with “patient-tailored” MAP (comparable to pre-operative MAP of the single patient). The study showed no differences in terms of major cardiac and neurologic events (11.7 and 12.6% respectively). However, also this study included only patients undergoing CABG surgery. However, all the these investigations showed conflicting evidences, without achieving a real consensus on what should be the most adequate MAP target during CPB.

The purpose of our randomized study is to define the best strategy to obtain an “ideal MAP” during CPB, comparing the historically accepted “standard MAP” (50–60 mmHg) with the “high MAP” (70–80 mmHg) and with the “patient-tailored MAP”.

To investigate the risk of hypoperfusion and to evaluate whether the MAP during CPB is adequate to avoid tissue hypoxia, the serum lactate value, as index of tissue anaerobiosis/hypoperfusion, will be collected at different timepoints [15–18]. To date, several studies have analysed the trend of lactates intraoperatively and postoperatively, and hyperlactatemia demonstrated to be a predictive factor of postoperative mortality and morbidity [19]. In particular, Demers et al. [20] described how a peak of lactates >4 mmol/l during CPB relates to postoperative mortality. Ranucci et al. [21] confirmed that hyperlactatemia >3 mmol/l during CPB relates to postoperative major complications. However, both studies did not give information about the precise value of lactates reached, neither about the entire trend of lactates released during CBP. Therefore, differently from previous studies, the primary endpoint of this trial will be the peak value (absolute value) of serum lactates (Lmax) measured during CPB.

### Objectives {7}

#### Primary objective

To assess the best CPB management strategy in order to obtain the patient’s “ideal MAP,” comparing the “standard MAP” (50–60 mmHg) versus the “high MAP” (70–80 mmHg) versus the “patient-tailored MAP” (comparable to the preoperative mean pressure of the single patient). The measurement of blood lactate leakage during CPB will be used as index of hypoxia. Then, we will be able to compare the three strategies of MAP in terms of tissue perfusion.

#### Secondary objectives

To assess the intraoperative outcomes, through the following perfusion parameters:

- Intraoperative cerebral perfusion (through monitoring of the NIRS values)
- Intraoperative pulmonary perfusion (through Pa/FiO_2_ ratio, paO_2_, paCO_2_ at blood gas analysis, and VO_2_R and DO_2_ at CDI® Blood Parameter Monitoring System 550)

To assess postoperative clinical outcomes, through the following parameters:

- Intraoperative and postoperative low cardiac output syndrome (according to the values of vasoactive inotropic score, VIS) [22]
- Postoperative cardiac function (measuring left ventricular ejection fraction, LVEF)
- Postoperative respiratory failure (through the values of Lung Injury Score, LIS, scale) [23]
- Postoperative stroke and entity of transitory or permanent neurological damage (through score of modified Rankin Scale—mRS) [24]
- Postoperative acute kidney injury (through AKI score)
- Postoperative hepatic failure (measuring the hepatic function and coagulation indices)
- Postoperative gastrointestinal ischaemia
- Postoperative cardiovascular death, or death due to other causes

To assess clinical outcomes at 30 days from surgery:

- Stroke and entity of the transitory or permanent neurologic damage (mRS score)
- Acute kidney injury (AKI score)
- Cardiac function (LVEF)
- Re-hospitalization
- Death for cardiovascular or other causes

#### Trial design {8}

The present study is an exploratory randomized controlled three parallel-group trial. The patient allocation is 1:1:1. The outcome of patients undergoing cardiac surgery and treated with a MAP standard during CPB is compared to patients treated with high MAP and patient-tailored MAP during CPB.

## Methods: participants, interventions and outcomes

### Study setting {9}

Coordinator Centre: Division of Cardiac Surgery, Azienda Ospedaliera Universitaria Integrata Verona, Italy.

Participating Centres:

Centre 1: Division of Cardiac Surgery at Ospedale Maggiore, Parma, Italy

Centre 2: Division of Cardiac Surgery at Azienda Sanitaria Universitaria Friuli Centrale, Udine, Itay

Centre 3: Division of Cardiac Surgery at Hospital Clinic de Barcelona, Barcelona, Spain

The study population includes all patients undergoing elective cardiac surgery. Enrolment and subsequent data collection will be performed in each participating centre. Statistical analyses will be performed by the Clinic Research Unit, University Hospital of Verona, Italy.

### Eligibility criteria {10}

#### Inclusion criteria

- Age > 18 years and < 80 years
- Elective surgery
- Index of surgical risk EuroSCORE II < 9%
- The following procedures will be considered: Isolated or combined with aortic or mitral valve surgery coronary artery bypass graft surgery for acute or chronic coronary artery disease isolated aortic valve replacement for aortic stenosis and/or aortic regurgitation; isolated mitral valve repair or replacement for mitral stenosis and/or mitral regurgitation; isolated ascending aorta surgery with or without aortic valve replacement
- Surgical approach through complete and/or mini sternotomy
- Preserved or mildly reduced left ventricular ejection fraction (LVEF ≥ 40%) at preoperative echocardiography.
- Patients with an estimated glomerular filtration rate (eGFR) ≥ 40 ml/min/mq calculated using the Modification of Diet in Renal Disease formula (MDRD)
- Signed informed consent

#### Exclusion criteria

- Age < 18 years and >80 years
- Reoperation
- Emergent, urgent and salvage procedures
- EuroSCORE II > 9%
- Right thoracotomy approaches
- Any surgical procedure not listed above (i.e. tricuspid valve surgery, aortic root surgery, congenital heart diseases, surgery necessitating hypothermic circulation arrest, surgical ablation of atrial fibrillation, etc.)
- More than mild left ventricular dysfunction at preoperative echocardiogram (LVEF < 40%)
- Patients with critical preoperative state: any ventricular fibrillation or ventricular tachycardia, preoperative cardiac massage, preoperative ventilation before anaesthetic room, preoperative inotropes or mechanical circulatory support planned before cardiac intervention (i.e. during coronary angiography) and other conditions according to EuroSCORE II definition.
- Patients with an estimated eGFR < 40 ml/min/mq calculated using the MDRD or patients on dialysis.
- Patients with chronic obstructive pulmonary disease > 3 stage according to Global Initiative for Chronic Obstructive Lung Disease (GOLD) 2019 classification.
- Patients with severe preoperative hepatic failure (CHILD-PUGH ≥ B)
- Patient with severe symptomatic carotid atheromasia

### Who will take informed consent? {26a}

Participants will be asked by the research team to provide informed consent for study participation.

### Additional consent provisions for collection and use of participant data and biological specimens {26b}

No biological specimen will be preserved for future analysis.

## Interventions

### Explanation for the choice of comparators {6b}

“MAP standard” is the control group because the value of 50–60 mmHg has been historically considered the MAP “standard of care” during CPB at the Division of Cardiac Surgeries of all Participating Centres.

### Intervention description {11a}

The patients enrolled to elective cardiac surgeries will be evaluated during a pre-operative outpatient visit and widely informed about the chance to participate in the study. When patients undergo to the elective surgery general anaesthesia is administered and CPB is installed. The nominal flow for each patient will be 2.4 l/min/m^2^.

A continuous monitoring of blood pressure will be performed invasively by cannulating the radial or femoral artery, as is customary in cardiac surgery.

To keep the MAP values around those corresponding to the randomized group, vasodilator (if MAP overcomes the assigned MAP value) or vasoconstrictor drugs will be used (if MAP value is lower than the assigned group). The following drugs will be used: nitroglycerine at incremental dose of 0.01 mcg/kg/min for a vasodilator effect and norepinephrine at incremental dose of 0.01 mcg/kg/min for a vasoconstrictor effect. The treatment groups will be:

*Group 1: Standard MAP*: MAP values between 50 and 60 mmHg as control group.

*Group 2: High MAP*: MAP values between 70 and 80 mmHg.

*Group 3: Patient-tailored MAP*: MAP comparable to the patient’s pre-operative MAP. This one will be calculated by performing 3 blood pressure measurement in three different moments of the day before surgery (at 8 am, at 3 pm and at 9 pm), and will be calculated using the standard formula “Diastolic AP + 0.33 × (systolic AP − Diastolic AP)”. The preoperative MAP value obtained will be target during CPB, within a range of ± 10 mmHg.

## Procedures

### Preoperative

- Preoperative outpatient visit (7 days before surgery)

- Clinical evaluation of the patient and eligibility for the study through collection of anamnestic data and physical examination.

- Day before surgery:

- Collection of informed consent for participating the study
- Randomization
- Measurement of preoperative MAP in every patient enrolled, regardless of the assignment group through randomization.
- Blood samples to assess: haemoglobin (Hb), haematocrit (Ht), white blood cells (WBC), platelets (Plt), C reactive protein (CRP), lactate dehydrogenase (LDH), prothrombin time (PT/INR), activated partial thromboplastin time (aPTT), fibrinogen and creatinine (mg/dl), lipase, pancreatic amylase, alanine aminotransferase (ALT), aspartate aminotransferase (AST), total bilirubin (Bil Tot), conjugated bilirubin (direct Bil), unconjugated bilirubin (indirect Bil), gamma-glutamyltransferase (GGT), alkaline phosphatase (ALP) and albumin
- Arterial blood gas test (ABG) to evaluate preoperative lactatemia
- Calculation of eGFR (according to MDRD) to evaluate preoperative renal function
- Evaluation of preoperative mRS in case of stroke in amnestic history.

- Intraoperative (postoperative day, POD, 0)

- A continuous monitoring of blood pressure will be performed invasively by cannulating the radial or femoral artery, as is customary in cardiac surgery. Data of MAP will be recorded at the beginning of CPB, every 20 min until minute 300 and at the end of CPB.
- ABG after orotracheal intubation, at the beginning of CPB, every 20 min during CPB (eventually until minute 300), at the end of CPB, at the end of surgery. Data collected: lactates as organ perfusion index, Hb and Hct.
- Real-time and continuous monitoring of respiratory gases during CPB suggestive of the perfusion trend, through CDI ® 550 Blood parameter monitoring system (Terumo Europe) [25], data collection related to oxygen delivery (DO_2_), oxygen consumption (VO_2_) and oxygen extraction (O_2_ER), at the beginning of CPB, every 20 min (until minute 300) and at the end of CPB.
- Intraoperative monitoring of Near-Infrared Spectroscopy NIRS [26] defined as non-invasive measurement of cerebral microcirculatory blood flow. Data will be collected at anaesthesia induction, before skin incision, at the beginning of CPB, every 20 min (until minute 300), at the end of CPB and at the end of surgery.
- Monitoring of CPB and aortic cross-clamp times
- Calculation of “vasoactive inotropic score” (VIS) [22] which relates the entity of inotropic and or vasoactive support. VIS max is obtained through the following calculation: [Dopamine dose (mcgkg/min)+ Dobutamine dose (mcg/kg/min) + 100 × Epinephrine dose (mcg/kg/min) + 50 × Levosimendan dose (mcg/kg/min) + 10 × Milrinone dose (mcg/kg/min) + 10,000 × Vasopressin dose (units/kg/min) + 100 × Norepinephrine dose (mcg/kg/min)]. The VIS will be calculated according to the length of surgery.
- Monitoring of nitroglycerine dose during surgery

### Postoperative

- POD at arrival in intensive care unit (ICU), POD 1 and 4

- Blood chemistry samples
- Calculation of eGFR (according to MDRD)
- ABG at arrival in ICU, at 3–6–12–24 h after surgery. Data collection on serum lactates, pH, paO_2_, paCO_2_, BE, HCO_3_−, calculation of the Pa/Fi ratio
- Chest X-ray and evaluation of eventual pulmonary damage through Murray “Lung Injury Score” (LIS) [23]. The score considers 4 criteria for the development of ALI/ARDS: hypoxemia, respiratory system compliance, chest radiographic findings and the positive expiratory pressure level. Each criteria receives a score from 0 to 4 according to the gravity of the condition. The final score is obtained dividing the collective score by the number of components used. A score equal to 0 shows the absence of pulmonary damage, a score between 1 and 2.5 shows a mild to moderate pulmonary damage and a final score major than 2.5 shows the presence of ARDS.
- Monitoring of estubation time (hours)
- Calculation of VISmax
- Monitoring of postoperative cardiac function at POD 4 through echocardiogram and evaluation of LVEF
- Monitoring of postoperative stroke; a diagnosed stroke by a neurologist according to clinical, radiological (CT scan and/or MRI) and electrophysiological examination (EEG). Patients affected by stroke will undergo “modified Rankin Scale” (mRS) calculation which evaluates the degree of disability following a stroke and will be compared to the preoperative mRS. The score goes from a minimum score of 0 (no disability) to 6 (death).
- Evaluation of acute kidney injury according to the definition of “Acute Kidney Injury” (AKIN) [27] through a stratification of renal damage in three stages: [1] Creatinine × 1.5–2.0 from baseline or Creatinine increased by at least 0.3 mg/dl (26.5μmol/L) [2]; Creatinine × 2.0–2.9 from baseline [3]; Creatinine > 3.0 from baseline or Creatinine increased at least 4 mg/dl (353.6 μmol/l) or the initiation of dialysis.
- Monitoring of gastrointestinal ischaemia: a diagnosed ischaemia by a surgery consultant according to the value of serum lactate, WBC, clinical and radiological (CT scan and/or abdomen ultrasound) examination.
- Monitoring of in-hospital death

- Follow-up visit at 30 days from surgery:

- Stroke and eventual transitory or permanent neurological damage (mRS score)
- Acute kidney injury (eGFR and AKIN score)
- Cardiac function (LVEF)
- Re-hospitalization
- Death for cardiovascular or other causes.

All the procedures listed above are considered the standard of care in the clinical practice at the Cardiac Surgery Divisions of all the Participating Centres.

### Criteria for discontinuing or modifying allocated interventions {11b}

If any of the following clinical situations occur during the study period in patients already enrolled, this will be reason for exclusion of the patient from the study: withdrawal of consent by the patient, unplanned additional procedures, necessary during surgery for complications in itinere (not provided in the preoperative planning), the need of mechanical circulatory support > 72 h.

### Strategies to improve adherence to interventions {11c}

Not applicable. Participation in the study does not require any change in usual care pathways and these will be maintained for all the study arms.

### Relevant concomitant care permitted or prohibited during the trial {11d}

Not applicable. Participation in the study does not require any change in usual care pathways and these will be maintained for all the study arms.

### Provisions for post-trial care {30}

There is no anticipated harm and compensation for study participation.

### Outcomes {12}

#### Primary outcome

The primary endpoint of the study is the serum lactate peak (Lmax) (mmol/l) detected during CPB. The mean of this value will be compared between the three groups of treatment.

#### Secondary outcomes

- The area under the curve (AUC) of the serum lactate values measured during CPB
- Number of cases with serum lactate peak > 3 mmol/l during CPB
- Evaluation of intraoperative cerebral perfusion (through monitoring of NIRS)
- Intraoperative pulmonary perfusion (through Pa/Fi ratio, paO_2_, paCO_2_ at ABG and VO_2_R and DO_2_ of CDI)
- Evaluation of intraoperative and postoperative low cardiac output syndrome (through the calculation of VISmax)
- Postoperative and 30-day LVEF (%)
- Evaluation of pulmonary injury (through the LIS)
- Postoperative and 30-day Acute Kidney Injury (according to AKIN score)
- Postoperative gastrointestinal ischaemia
- Hepatic function and coagulation indexes
- Evaluation of neurological dysfunction (as dichotomous variable) and evaluation through mRS (0–6) in case permanent neurological injury
- In-hospital mortality and at 30 days from surgery
- Re-hospitalization

### Participant timeline {13}

**Fig. 1:**
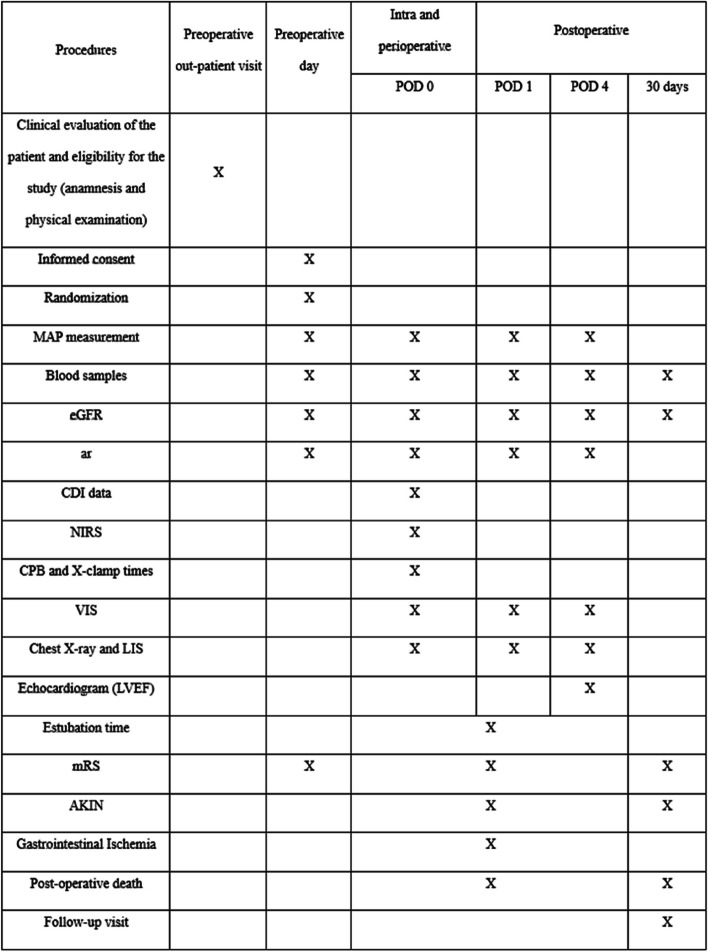
Flowchart of the study

The patient will participate to the study from the preoperative day (when he/she will sign the informed consent) until the 30th after surgery. Figure 1 shows all the interventions and data collection timeline.

### Sample size {14}

The sample size has been calculated for the primary endpoint: the maximum serum lactate value (Lmax). A preliminary analysis was performed on a sample size of 128 consecutive patients undergone to elective cardiac surgery at Cardiac Surgery Division at AOUI of Verona (about the 10% of the annual volume cases at our Institution). All patients were treated with standard MAP (standard of care) and the estimated mean of Lmax during CPB was 1.25 mmol/l, with a standard deviation of 0.7 mmol/l. Starting from this value, a reduction of −15% of the mean Lmax was considered clinically significant for every comparison that will be performed in the study, as listed below:

1. Standard MAP vs High MAP (−15%) difference *d*=−0,19
2. Standard MAP vs patient-tailored MAP (−15%) difference *d*=−19
3. High MAP vs patient-tailored MAP (−15%) difference *d*=−19

Based on these assumptions, performing the Mann-Whitney non-parametric test and considering an alpha of 0.05 s. Bonferroni for all the three comparisons (corrected Alpha= 0.01667) and a power of 80%, 300 patients should be enrolled for each group, for a total of 900 patients (PASS 14). To account for a potential drop-out of 10%, the size becomes 333 patients per group, then a total of 999 patients. The number of patients that each Participating Centres should recruit has been calculated based on the annual cases volume of each Centre, as follows:

- Cardiac Surgery Division of AOUI Verona: 327 cases (109 per group)
- Cardiac Surgery Division of Ospedale Maggiore di Parma: 162 cases (54 per group)
- Cardiac Surgery Division of Azienda Sanitaria Universitaria Friuli Centrale di Udine: 231 cases (77 per group)
- Cardiac Surgery Division of Hospital Clinic de Barcelona, Spain: 279 cases (93 per cases)

### Recruitment {15}

Patients will be recruited at Cardiac Surgery Division of each Participating Centre. Every patient fulfilling the eligibility criteria, after the signature of the informed consent, will be enrolled and randomized to one of the three groups of treatment. Recruitment will continue until the expected total number of patients is reached.

## Assignment of interventions: allocation

### Sequence generation {16a}

The list of randomization will be generated for each centre using STATA statistical software 14, by the Clinical Research Unit of Azienda Ospedaliera Universitaria Integrata of Verona. The balanced block randomization method will be used. To reduce predictability of a random sequence, details of any planned restriction will be provided in a separate document that is unavailable to those who enrol participants or assign interventions

### Concealment mechanism {16b}

Use of a validated password website will ensure concealment.

### Implementation {16c}

After a first researcher takes the informed consent forms, a second researcher will use the list of randomization to allocate the patient to one of the study arms. The study group will be revealed at the same time to the patient, the researchers, the first operator and the operating room staff.

## Assignment of interventions

### Who will be blinded {17a}

The researcher at the time of the informed consent and the data analyst will be blinded. Data is e-data so there are no outcome assessors.

### Procedure for unblinding if needed {17b}

Randomization is communicated to the patients and to the investigators.

## Data collection and management

### Plans for assessment and collection of outcomes {18a}

All preoperative, intraoperative and postoperative procedures are standard of care in each Cardiac Surgery Division. The data collection will take place during the patient hospitalization by the research team in a dedicated datasheet. All the data are listed in the protocol and will be retrieved from the Hospital health information system. A separated datasheet with the same coding will be used for the participating centres. At the end of the study, all datasheets will be unified in a unique database and analysed by the promoting centre.

### Plans to promote participant retention and complete follow-up {18b}

The 30-day outpatient visit will be planned at the time of patient’s discharge.

### Data management {19}

All data will be retrieved from electronic patient records at the time of hospitalization and then at the follow-up visit and collected in a dedicated anonymized datasheet. Informed consent and end-of-trial dates will be recorded in the electronic patient dossier, and signed paper forms will be stored within each hospital in a locked room, whose keys will only be available to the research team and will be safeguarded by the principal investigator.

### Confidentiality {27}

Research data will be stored using a study identification code for each participant. The key to the identification code list will only be available to the research team during the study and will be documented and safeguarded by the principal investigator according to research guidelines after completion of the study. No patient identification details will be reported in publications.

Data collection and management will be in accordance with the EU regulation 2016/679, the Privacy Code (D.lgs 196/03 s.m.i) and Guide Lines of 24th July 2008, and will be guaranteed by the promoter of the study. The promoter will store the original paper documentation (i.e. informed consent) for at least 7 years in compliance with LD 200/2007.

### Plans for collection, laboratory evaluation and storage of biological specimens for genetic or molecular analysis in this trial/future use {33}

Not applicable. Collection, laboratory evaluation and storage of biological specimens for genetic or molecular analysis are not included in the study.

### Statistical methods

#### Statistical methods for primary and secondary outcomes {20a}

Demographic and clinical characteristics will be presented as percentage in case of ordinal variables and as percentages, means, medians and standard deviations and/or interquartile range in case of categorical and/or quantitative variables. The Pearson *χ*^2^ and the *H*-test by Kruskal-Wallis will be used for any assessment of differences between the three groups. To evaluate the primary endpoint of the study, the Mann-Whitney test will be used for comparisons between groups with an alpha equal to 0.01667. The Mann-Whitney test will also be used to evaluate the secondary endpoint obtained by AUC, while the Pearson *χ*^2^ will be used to compare the proportion of cases above and below the serum lactate peak cut-off > 3mmol. To compare the secondary endpoints in the three groups according to the types of variables, ANOVA or non-parametric *H*-test by Kruskal-Wallis will be used for quantitative variables, while the Pearson *χ*^2^ test will be performed for dichotomous or categorical variables. ANOVA for repeated measures, or Friedman test or the mixed effects model will be used for variables collected at different timepoints. A *p*-value < 0.05 will be considered statistically significant. Both an intention to treat (ITT) and per protocol (PP) analysis will be performed.

#### Interim analyses {21b}

There are no interim analyses planned.

#### Adverse events {22}

There are no adverse events or serious adverse events expected other than those of routine cardiothoracic surgery. However, all data will still be collected in the dataset and eventually communicated to the Ethical Committee of “Province di Verona e Rovigo.”

#### Methods for additional analyses (e.g. subgroup analyses) {20b}

There are no subgroup analyses planned.

#### Methods in analysis to handle protocol non-adherence and any statistical methods to handle missing data {20c}

Missing data will be handled by different data imputation methods (IPW-inverse probability weighting and LCOF-Last-Observation-Carried-Forward method) with sensitivity analysis.

#### Plans to give access to the full protocol, participant-level data and statistical code {31c}

The data set and statistical code analysed as part of the current study will be available by the corresponding author upon reasonable request, as is the full protocol.

## Oversight and monitoring

### Composition of the coordinating centre and trial steering committee {5d}

The promoter will have the responsibility for the study management. The study executive committee will be constituted by the principal investigator of the research teams of each participating centre. The executive committee will be responsible for the daily running of the trial. The trial steering committee will be constituted by the promoter and the principal investigators of the participating centres who will meet twice a year to monitor the conduct of the study.

### Composition of the data monitoring committee, its role and reporting structure {21a}

The data monitoring committee will be constituted by the promoter and the principal investigators of the participating centres who will meet twice a year to check the appropriateness of the investigation, by checking the data of a minimum of 40% of the patients.

### Adverse event reporting and harms {22}

All the adverse events, even if not related to the MAP during CPB, but occurring during the study, will be registered in the datasheet.

### Frequency and plans for auditing trial conduct {23}

Regular meetings (every 6 months) will be held to monitor the study conduct and address potential problems.

### Plans for communicating important protocol amendments to relevant parties (e.g. trial participants, ethical committees) {25}

Any changes to the study protocol will be communicated to the AOUI of Verona Institutional Review Board and the Regional Ethical Committees responsible for approving the study.

### Dissemination plans {31a}

#### Dissemination policy

The data will be property of AOUI of Verona (no profit and no sponsor study) and the data obtained will be presented at international meetings and will be submitted at international scientific journals with IF. A position of co-authorship will be reserved to the biostatisticians involved in the study. The investigators will be eligible for authorship if they contribute to the data collection, the analysis and interpretation of the data, the writing and critical review of the manuscripts. Two authors per centre will be included as authors of the study. When a paper is submitted to a Journal with a maximum number of co-authors, the Directive Committee will establish the authors according to their contribution to the design of the study, the data collection, the interpretation of data, the writing and the critical review of the paper.

## Discussion

This randomized controlled trial is designed to investigate the MAP management during CPB in cardiac surgery, since it remains one of the “grey zone” of cardiac surgery. Providing and adequate tissue perfusion is the main goal of CPB during cardioplegic arrest. Nonetheless, some patients still reported ischaemic complication after heart operations. To date, only single-centre studies have been reported with small sample sizes, selected populations and contradictory findings. We started recruiting the first patients in 2021; however, during the first years of the study, we encountered some practical problems. Due to supply issues, we were without access to the CDI monitoring for a long period, causing us to slow down patient recruitment based on the availability of monitoring equipment. Additionally, it was not always easy to maintain the pressure within the range established by the randomization group, due to specific alterations in the arterial resistance of the patients (e.g. deep vasoplegia in “high MAP” or hypertension in “standard MAP”). So far, two patients have dropped out of the study for this reason. However, this trial is the first prospective multicentre randomized controlled study that compare three strategies of MAP management during CPB using the serum lactate peak as primary endpoint. Finding the most adequate approach will contribute to improve the outcome of patients undergoing heart surgery.

### Trial status

Protocol version 2, date approval: 15/01/2021. First recruitment date: 02/05/2021. The approximate date when recruitment will be completed: 31/12/2026.

## Abbreviations

AAR: Ascending aorta replacement
ABG: Arterial blood gas test
ALI: Acute lung injury
AP: Arterial pressure
ARDS: Acute respiratory distress syndrome
AUC: Area under the curve
BE: Base excess at hemogas analysis
BSA: Body surface area
COPD: Chronic obstructive pulmonary disease
CABG: Coronary artery bypass graft
CPB: Cardiopulmonary bypass
DO_2_: Oxygen delivery
FiO_2_: Oxygen inspired fraction
eGFR: Estimated glomerular filtration rate
GOLD: Global Initiative for Chronic Obstructive Lung Disease
HCO3-: Ion bicarbonate concentration
ICU: Intensive care unit
LIS: Lung Injury Score
LVEF: Left ventricular ejection fraction
Lmax: Maximum level of lactate
MAP: Median arterial pressure
MDRD: Modification of Diet in Renal Disease
MMSE: Mini-Mental State Examination
mRS: Modified Ranking Scale
NIRS: Near-infrared spectroscopy
O2Er: Oxygen extraction
PaO_2_: Partial pressure of oxygen
paCO_2_: Partial pressure of carbon dioxide
POD: Postoperative day
XR: X-ray
VIS: Vasoactive inotropic score
VO2: Oxygen consumption
